# Multidimensional Geriatric Prognostic Index, Based on a Geriatric Assessment, for Long-Term Survival in Older Adults in Korea

**DOI:** 10.1371/journal.pone.0147032

**Published:** 2016-01-15

**Authors:** Hee-Won Jung, Jin Won Kim, Ji Won Han, Kayoung Kim, Jee Hyun Kim, Kwang-Il Kim, Cheol-Ho Kim, Ki Woong Kim

**Affiliations:** 1 Division of Geriatrics, Department of Internal Medicine, Seoul National University Bundang Hospital, Seoul National University College of Medicine, Seongnam, Korea; 2 Division of Hematology and Medical Oncology, Department of Internal Medicine, Seoul National University Bundang Hospital, Seoul National University College of Medicine, Seongnam, Korea; 3 Department of Neuropsychiatry, Seoul National University Bundang Hospital, Seongnam, Korea; 4 Department of Psychiatry, Seoul National University College of Medicine, Seoul, Korea; 5 Department of Brain and Cognitive Science, Seoul National University College of Natural Sciences, Seoul, Korea; UNIFESP Federal University of São Paulo, BRAZIL

## Abstract

The patient´s survival estimate is important for clinical decision-making, especially in frail patients with multimorbidities. We aimed to develop a multidimensional geriatric prognosis index (GPI) for 3- and 5-year mortality in community-dwelling elderly and to validate the GPI in a separate hospital-based population. The GPI was constructed using data for 988 participants in the Korean Longitudinal Study on Health and Aging (KLoSHA) and cross-validated with 1109 patients who underwent a geriatric assessment at the Seoul National University Bundang Hospital (SNUBH). The GPI, with a total possible score of 8, included age, gender, activities of daily living, instrumental activities of daily living, comorbidities, mood, cognitive function, and nutritional status. During the 5-year observation period, 179 KLoSHA participants (18.1%) and 340 SNUBH patients (30.7%) died. The c-indices for 3- and 5-year mortality were 0.78 and 0.80, respectively, in the KLoSHA group and 0.73 and 0.80, respectively, in the SNUBH group. Positive linear trends were observed for GPI scores and both 3- and 5-year mortality in both groups. In conclusions, using common components of a geriatric assessment, the GPI can stratify the risk of 3- and 5-year mortality in Korean elderly people both in the community and hospital.

## Introduction

The global population is aging and the proportion of older adults is increasing in South Korea [1]. The patient’s survival estimate is important for individualized decision-making, especially in frail patients with multimorbidities [2, 3].

Frailty is associated with increased mortality [4, 5] and vulnerability to surgery and chemotherapy for cancer [6, 7]. Furthermore, frailty is closely associated with multimorbidities and functional impairment [8, 9]. Although frailty is correlated with chronological age, there are inter-individual differences that must be considered [4, 5]. Geriatric assessment (GA) has the ability to assess the level of frailty in individual patients [5]. Therefore, when estimating the survival of older adults, a GA, which includes the characteristics of frailty, can be beneficial for personalized decisions regarding treatments that might result in complications.

A number of short-term [10, 11] and long-term [12] prediction models for mortality have been developed in various settings in different countries [13]. However, there are some quality barriers including potential for bias, compatibility in another population, and accuracy to use routinely in general practice [13]. Because specific data acquisition is difficult, practical issues remain for routinely adaptation.

Therefore, we aimed to develop a practical and generally accepted multidimensional geriatric prognosis index (GPI) based on GA to predict long-term mortality in Korean community-dwelling elderly and validate the GPI in a separate hospital-based population.

## Materials and Methods

### Study design and population

This study included two populations: community-based prospective cohort and hospital-based retrospective cohort.

The community-based population was based on the dataset from the Korean Longitudinal Study on Health and Aging (KLoSHA), which included people aged ≥65 years old in the city of Seongnam city [14]. For the derivation of the GPI, we used the final dataset of baseline data from KLoSHA, which included 721 randomly sampled people ≥ 65 years old and 278 people ≥ 85 years old who voluntarily participated. The baseline evaluation was performed at the Seoul National University Bundang Hospital (SNUBH) from 2005 to 2006.

For the hospital-based cohort, we reviewed the medical records of 1282 patients ≥60 years old who underwent a GA in the outpatient geriatric clinic or inpatient wards of the SNUBH between 2004 and 2007.

### Measurements and definitions

Variables for the GPI were selected in an *a priori* fashion to facilitate adaptation for a widespread GA with generally accepted domains and to prevent over-fitting in the study dataset; these variables included age, gender, activities of daily living, instrumental activities of daily living, comorbidities, mood, cognitive function, and nutritional status, which are associated with prognosis.

For these variables, the KLoSHA used the Korean Activities of Daily Living (K-ADL) [15], Korean Instrumental Activities of Daily Living (K-IADL) [15], Cumulative Illness Rating Scale for Geriatrics (CIRS-G) [16], Korean version of the Geriatric Depression Scale (GDS) [17], and Nutrition Screening Initiative (NSI) [18]. At the SNUBH, the modified Barthel index [19], Lawton and Brody Index [20], Charlson’s comorbidity index (CCI) [21, 22], Korean version of the Geriatric Depression Scale Short Form (GDS-SF) [23], and Mini Nutritional Assessment (MNA) [24] were used. The Korean Mini-Mental State Examination (MMSE) [25] was used both in the KLoSHA and at SNUBH.

Because the GAs in the KLoSHA and at SNUBH used different measures for each domain, we created a method to categorically interpret each domain (Table 1). The coding and cutoff values were determined based on the effects of the GA domains on mortality that were observed in a previous study [5]. We tried to simplify the coded scores from the many tools (i.e., 0, 0.5, or 1) to facilitate the comparability between the GA domains from the KLoSHA and SNUBH datasets. The final score of the GPI was calculated by summing the scores from age, gender, and the 6 GA domains, resulting in a possible score ranging from 0 to 8.

**Table 1:** Scoring of geriatric assessment variables included in the geriatric prognosis index.

Variables	Score
Age (years)	
65–74	0
75–84	0.5
≥85	1
Gender	
Female	0
Male	1
ADL	
Independent	0
Minimally dependent (1 domain impaired)	0.5
Dependent (≥2 domains impaired)	1
IADL	
Independent	0
Minimally dependent (1 domain impaired)	0.5
Dependent (≥2 domains impaired)	1
Comorbidity	
CCI 0 (or CIRS-G 0–3)	0
CCI ≥1 (or CIRS-G ≥4)	1
Mood	
GDS ≤13 (or GDS-SF ≤4)	0
GDS 14–21 (or GDS-SF 5–8)	0.5
GDS 22–30 (or GDS-SF 9–15)	1
Cognition	
MMSE 25–30	0
MMSE 18–24	0.5
MMSE ≤17	1
Nutritional status	
MNA 24–30 (or NSI 0–2)	0
MNA 17–23.5 (or NSI 3–5)	0.5
MNA ≤16.5 (or NSI ≥6)	1

ADL, activities of daily living; IADL, instrumental activities of daily living; CCI, Charlson’s comorbidity index; CIRS-G, Cumulative Illness Rating Scale for Geriatrics; GDS, Korean version of the Geriatric Depression Scale; GDS-SF, Korean version of the Geriatric Depression Scale Short Form; MMSE, Korean Mini-Mental State Examination; MNA, Mini Nutritional Assessment; NSI, Nutrition Screening Initiative

### Study outcomes

The survival status, including cause and date of death, was acquired from the National Statistical Office of Korea for all of the participants in the development (KLoSHA) and validation (SNUBH) cohorts as of December 2011 (medial follow-up, 68.8 months) and December 2013 (median follow-up, 71.0 months), respectively. To analyze the 3- and 5-year mortality rates, participants who were alive at 3 and 5 years from the baseline GA were censored. Also, to remove the impact of acute illness on long-term mortality, we excluded participants who died within 6 months from the baseline GA (n = 11, development cohort; n = 173, validation cohort) from the final analysis.

### Statistical analysis

Because 196 of the 988 participants in the development cohort had at least 1 missing GA domain, we used a multiple imputation procedure with age, gender, K-ADL, K-IADL, CIRS-G, GDS, MMSE, and NSI. This procedure provided complete imputation for the 196 participants, resulting in a dataset of 988 participants. In the same manner, the data for 179 patients with at least 1 missing GA domain in the validation cohort underwent a multiple imputation procedure with age, gender, modified Barthel index, Lawton and Brody Index, CCI, GDS-SF, MMSE, and MNA.

The baseline characteristics were compared using independent *t*-tests for continuous variables and Chi-square tests for dichotomized variables. Linear regression analysis was used to evaluate associations between age and GPI. To analyze mortality, the predicted 3- and 5-year probability of mortality based on the GPI was calculated using a logistic regression model with GPI as the linear term in the development cohort, and the observed mortality in the validation cohort was compared with this prediction. The 95% confidence intervals (CIs) for the expected and observed mortality were calculated using a binomial distribution. To validate the ability of the GPI to predict 3- and 5-year survival, we used receiver operating characteristic analysis and calculated the c-index. The data for the development and validation cohorts were pooled for sensitivity analysis. Statistical analyses were conducted using STATA 12.0 (StataCorp, College Station, TX, USA)

### Ethics Statement

The study adhered to the guidelines of the Declaration of Helsinki, and the Institutional Review Board of the Seoul National University Bundang Hospital approved the study protocol (B-1211/178-112). Written informed consent was acquired from all of the KLoSHA participants, and the need for informed consent was waived by the Institutional Review Board for the retrospective cohort from the Seoul National University Bundang Hospital (B-1211/178-112). Patient records/information was anonymized and de-identified prior to analysis for the retrospective cohort.

## Results

### Baseline characteristics

The development cohort included 988 participants, and the validation cohort included 1109 participants (Table 2). The validation cohort was older and had worse functional status, comorbidity scores, mood, cognition, and nutritional status than the community-based development cohort. The mean GPI score was also significantly higher (3.6) in the validation cohort than the development cohort (3.0).

**Table 2:** Baseline demographic and functional characteristics of the development (Korean Longitudinal Study on Health and Aging [KLoSHA]) and validation (Seoul National University Bundang Hospital [SNUBH]) cohorts, by geriatric assessment parameter.

Variables	KLoSHA (n = 988)	SNUBH (n = 1109)	*P*-value
Age (years)	75.8 ± 8.9	76.7± 6.8	0.015
Gender			
Male	431 (43.6)	402 (36.2)	0.001
Female	557 (56.4)	707 (63.8)	
ADL			
Independent	885 (89.6)	623 (56.2)	
Minimally dependent (1 domain impaired)	53 (5.4)	146 (13.2)	
Dependent (≥2 domains impaired)	50 (5.1)	340 (30.7)	<0.001
IADL			
Independent	462 (46.8)	477 (43.0)	
Minimally dependent (1 domain impaired)	153 (15.5)	92 (8.3)	
Dependent (≥2 domains impaired)	373 (37.8)	540 (48.7)	<0.001
Comorbidity			
CCI 0 (or CIRS-G 0–3)	489 (49.5)	366 (33.0)	
CCI ≥1 (or CIRS-G ≥4)	499 (50.5)	743 (67.0)	<0.001
Mood			
GDS ≤13 (or GDS-SF ≤4)	579 (58.6)	571 (51.5)	
GDS 14–21 (or GDS-SF 5–8)	277 (28.0)	266 (24.0)	
GDS 22–30 (or GDS-SF 9–15)	132 (13.4)	272 (24.5)	<0.001
Cognition			
MMSE 25–30	454 (46.0)	453 (40.8)	
MMSE 18–24	369 (37.3)	338 (30.5)	
MMSE ≤17	165 (16.7)	318 (28.7)	<0.001
Nutritional status			
MNA 24–30 (or NSI 0–2)	356 (36.0)	346 (31.2)	
MNA 17–23.5 (or NSI 3–5)	212 (21.5)	411 (37.1)	
MNA ≤16.5 (or NSI ≥6)	420 (42.5)	352 (31.7)	<0.001
GPI	3.0 ± 1.5	3.6 ± 1.9	<0.001

Data are presented as mean ± SD or n (%).

ADL, activities of daily living; IADL, instrumental activities of daily living; CCI, Charlson’s comorbidity index; CIRS-G, Cumulative Illness Rating Scale for Geriatrics; GDS, Korean version of the Geriatric Depression Scale; GDS-SF, Korean version of the Geriatric Depression Scale Short Form; MMSE, Korean Mini-Mental State Examination; MNA, Mini Nutritional Assessment; NSI, Nutrition Screening Initiative; GPI, geriatric prognostic index.

The GPI score was positively associated with age in both the development (B = 0.14, 95% CI 0.12–0.16, R^2^ = 0.43) and validation (B = 0.07, 95% CI 0.07–0.08, R^2^ = 0.28) cohorts in the linear regression analysis. Also, the GPI score was significantly higher in male than female in both the development (3.3 ± 1.3 vs. 2.7 ± 1.6, *P* < 0.001) and validation (4.0 ± 1.9 vs. 3.4 ± 1.8, *P* < 0.001) cohorts.

### Differences in 3- and 5-year mortality, estimated using the geriatric prognosis index

In the follow up, the mean period of observation was 63.4 +/- 15.4 months in the development cohort. Two hundred and ten (21.3%) participants died. Also, 92 (9.3%) participants died within 3 years, and 179 (18.1%) participants died within 5 years from the baseline evaluation. During the entire observation period of 65.6 ± 31.3 months in the validation cohort, 488 (44.0%) participants died. In addition, 225 (20.3%) participants died within 3 years, and 340 (30.7%) participants died within 5 years from the baseline evaluation.

In the logistic regression analysis with GPI as the linear term, a linear trend was observed in the 3-year mortality in both the development (odds ratio [OR] 2.0, 95% CI 1.7–2.4, *P* < 0.001) and validation (OR 1.6, 95% CI 1.4–1.7, *P* < 0.001) cohorts. The same trend was observed for 5-year mortality in both the development (OR 2.2, 95% CI 1.9–2.5, *P* < 0.001) and validation (OR 1.7, 95% CI 1.5–1.9, *P* < 0.001) cohorts.

**Fig 1:**
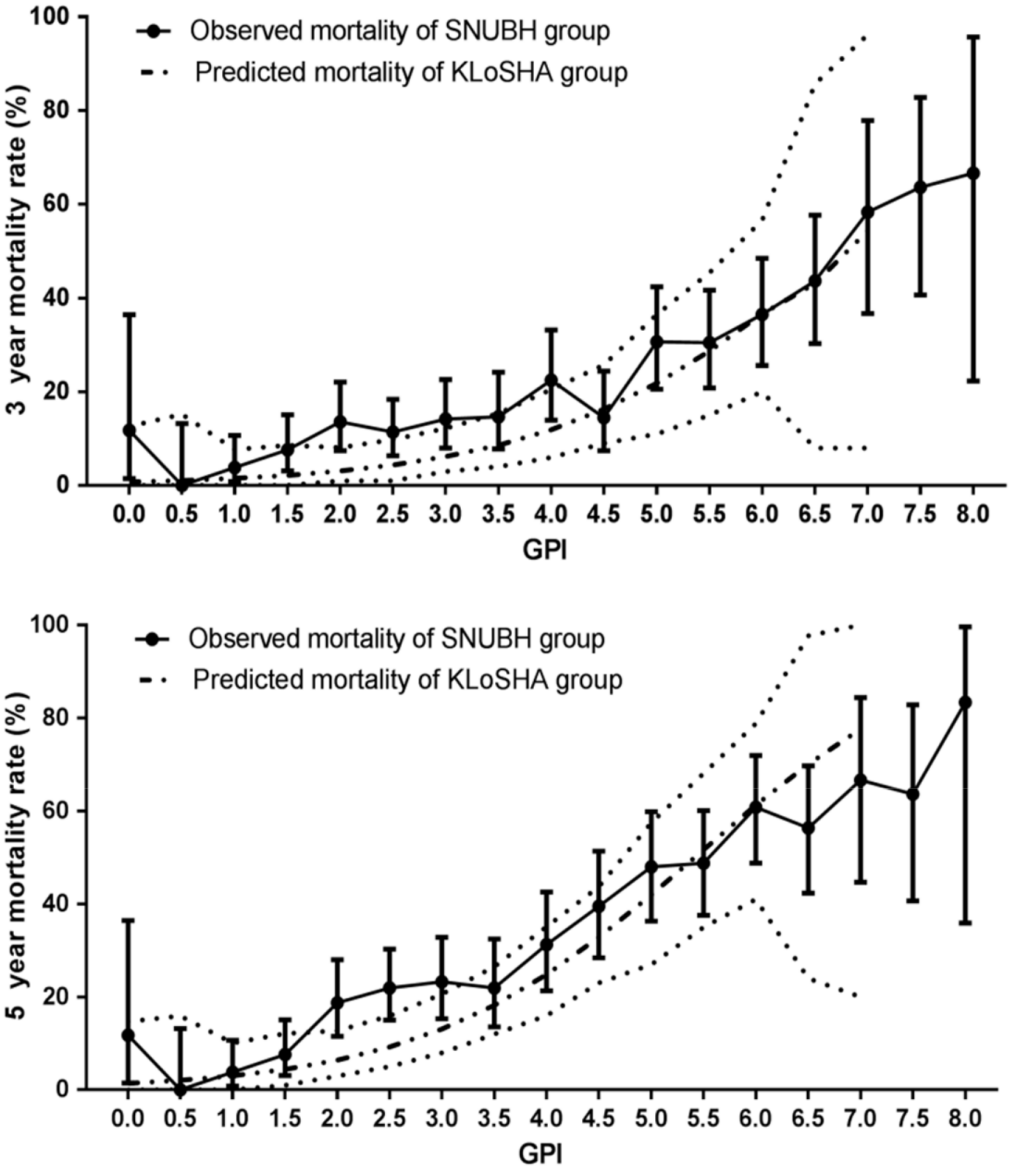
Comparisons of predicted 3- (A) and 5-year (B) mortality by geriatric prognostic index (GPI) score. Dotted lines show the 95% confidence interval. Predicted 3-year mortality (%) = 100 * exp(-4.903 + 0.726 * [GPI]) / (1 + exp(-4.903 + 0.726 * [GPI])); Predicted 5-year mortality (%) = 100 * exp(-4.247 + 0.784 * [GPI]) / (1 + exp(-4.247 + 0.784 * [GPI])) KLoSHA, Korean Longitudinal Study on Health and Aging; SNUBH, Seoul National University Bundang Hospital

The predicted 3- and 5-year mortality rates in the development cohort based on the logistic models in addition to the observed 3- and 5-year mortality rates in the validation cohort are shown in the S1 and S2 Tables. In Fig 1, the 3- and 5-year predicted mortality rates by GPI score in the development cohort are compared with the 3- and 5-year observed mortality rates in the validation cohort; in addition, the fitted equations for the 3- and 5-year mortality rates (%) are shown.

### Internal and external validation of the geriatric prognosis index

In the development cohort, the c-index of the GPI to predict 3-year mortality was 0.78 (95% CI 0.74–0.82), which was not significantly different (*P* = 0.757) from the c-index of the model including only age and gender (0.77, 95% CI 0.73–0.72). The c-index for predicting 5-year mortality with the GPI was 0.80 (95% CI 0.76–0.83), which was not significantly different (*P* = 0.639) from the c-index of the model using only age with gender (0.79, 95% CI 0.76–0.82).

In the validation cohort, the c-index of the GPI to predict 3-year mortality was 0.73 (95% CI 0.69–0.72), which was significantly higher (*P* = 0.028) than the c-index of the model including only age and gender (0.66, 95% CI 0.68–0.76). The c-index for predicting 5-year mortality with the GPI was 0.80 (95% CI 0.77–0.82), which was significantly higher (*P* < 0.001) than the c-index of the model using only age with gender (0.70, 95% CI 0.67–0.73).

### Sensitivity analysis

In the sensitivity analysis, the same linear trend in mortality based on the GPI was observed (3-year mortality, OR 1.6, 95% CI 1.5–1.7; 5-year mortality, OR 1.8, 95% CI 1.7–2.0). The c-index for the prediction of 3-year mortality was significantly higher (*P* = 0.001) for GPI (0.75, 95% CI 0.72–0.78) than for only age with gender (0.70, 95% CI 0.67–0.73). The c-index for the prediction of 5-year mortality was also significantly higher (*P* < 0.001) for GPI (0.77, 95% CI 0.75–0.79) than for only age with gender (0.72, 95% CI 0.70–0.75).

## Discussion

In the present study, multidimensional GPI was devised and validated, including mood, cognitive function, and nutritional status as well as function of daily activities, which was based on common GA domains, to predict long-term mortality in both community and hospital settings. Also, GPI could predict 3- and 5-year mortality with a significantly higher c-index than age combined with gender in a hospital-based population.

In previous studies, a number of prognostic indices were reported in various diseases and certain setting such as community-dwelling older adults, nursing home residents, and hospitalized older adults [11–14]. Pilotto index was developed to predict one-year mortality in hospitalized older patients [12]. Frailty index could also predict postoperative mortality [6]. However, because there are some quality barriers including potential for bias, compatibility in another population, and accuracy, these tools are not used routinely in general practice across patient groups that differ according to severity of diseases, data collection, location, and time. Furthermore, previous indices were developed from administrative data sets or the information that may not be routinely assessed in older patients [13]. Even if quality barriers are overcome, practical issues remain for routinely adaptation. In our study, the GPI was based on GA which could be tested routinely in practice for older patients. Variables for the GPI were selected in an *a priori* fashion to facilitate adaptation for a widespread GA with generally accepted domains and to prevent over-fitting in the study dataset. Therefore, our GPI could be more practical and fruitful than other tools.

The 5-year mortality rate of the community-dwelling elderly in the present study was similar to the 4-year and 5-year mortality in previous reports [12, 26], as was the predictability (c-index) of our model. In the hospital-based group, the mortality rate and GPI score were higher than in the community-based group, as expected. The c-index of the GPI to predict 3-year mortality and 5-year mortality was significantly higher in hospital-based population with more comorbidity and function decline than in community-dwelling elderly.

Because of the growing multimorbid and frail population, there are a higher number of elderly patients with cancer or cardiovascular disease requiring medical therapy or invasive procedures that could cause severe complications. In addition, the cost-effectiveness and futility of cancer screening or treatment strategies as well as therapeutic goal setting for chronic diseases are important issues in frail, multimorbid people [2, 27]. In a study that included Medicare beneficiaries [28], comorbidity status was a significant factor for life expectancy during cancer screening in elderly people, indicating that more than age should be considered for mortality. The inclusion of life expectancy might also be helpful to select patients who will benefit most from adjuvant or palliative chemotherapy for the treatment of colon, lung, and breast cancers, which are increasingly detected in older patients [29]. In the present study, a positive linear trend was observed between GPI and 3- and 5-year mortality in both the community and hospital-based populations. Therefore, clinicians could choose not to screen for cancer or use a more conservative regimen for adjuvant or palliative cancer treatment in this group. Also, health professionals involved in the multidisciplinary care of older people should be advised to prevent and manage deterioration in functional status.

There are several strengths of the present study. First, our GPI encompassed most geriatric factors including mood, cognitive function, and nutritional status as well as function of daily activities. We selected components that are usually found in a standardized GA. To facilitate the compatibility with other tools, we assigned a score for each domain rather than using the score directly from the tool. Second, although hazard ratios for mortality could be used to weight the GA domains in the development cohort, we did not use a weighting strategy to prevent over-fitting. Using this scoring method, the GPI was able to predict 3- and 5-year mortality in both community- and hospital-based populations, ensuring transportability of this index, which is required for the compatibility of a prognostic index in another population [13]. Third, we had access to survival data from the government for a relatively long period of time.

There are also certain limitations to the study. First, in the community-based population, the GPI performed similarly to the model with age and gender for predicting mortality. This finding could be explained by fewer comorbidities and better functional status in the community-based population. In a hospital-based population with worse functional status and more comorbidities, the GPI showed better performance and might be more appropriate. Second, we had to interpret and compare scores from the different tools that were used for each GA domain in each setting. However, the trends for mortality were similar in both settings. Third, the number of patients with similar GPI score might relatively too small for GPI to be generalized before more research would be done in the other dataset. Finally, the performance of this novel GPI was not directly compared with previous prognostic tools. The direct comparison was impossible in retrospective analysis because specific data were needed for each tool. However, this novel GPI is valuable, which showed high c-index to predict long-term mortality in both community and hospital settings.

In conclusion, a practical and generally accepted multidimensional GPI, which was developed in this study based on common GA components, stratified mortality in Korean elderly people. This index can provide information for clinicians regarding life expectancy and assist in individualized decision making for the treatment of an aging population.

## Supporting Information

S1 Table Observed and predicted 3-year mortality rate in the development cohort (Korean Longitudinal Study on Health and Aging [KLoSHA]) and observed 3-year mortality rate in the validation cohort (Seoul National University Bundang Hospital [SNUBH]), by geriatric prognostic index (GPI) score. (DOCX)

S2 Table Observed and predicted 5-year mortality rate in the development cohort (Korean Longitudinal Study on Health and Aging [KLoSHA)] and observed 5-year mortality rate in the validation cohort (Seoul National University Bundang Hospital [SNUBH]) group, by geriatric prognostic index (GPI) score. (DOCX)
