# Supplementary material for: Multidimensional Geriatric Prognostic Index, Based on a Geriatric Assessment, for Long-Term Survival in Older Adults in Korea
Source: PLoS One. 2016 Jan 15;11(1):e0147032. doi: 10.1371/journal.pone.0147032 (PMC4714804; doi:10.1371/journal.pone.0147032)
Supplement: S1 Table — (DOCX) [file pone.0147032.s001.docx]

S1 Table

|  | KLoSHA | | | | | SNUBH | | |
| --- | --- | --- | --- | --- | --- | --- | --- | --- |
| GPI score | Number | Observed mortality rate | 95% CI (%) | Predicted mortality rate (%) | 95% CI (%) | Number | Observed mortality rate | 95% CI (%) |
| 0 | 34 | 0 | 0.0 - 10.3 | 0.7 | 0.0 - 12.6 | 17 | 11.8 | 1.5 - 36.4 |
| 0.5 | 29 | 0 | 0.0 - 11.9 | 1.1 | 0.0 - 15.2 | 26 | 0 | 0.0 -13.2 |
| 1 | 75 | 0 | 0.0 - 4.8 | 1.5 | 0.0 - 7.7 | 79 | 3.8 | 0.8 - 10.7 |
| 1.5 | 76 | 1.3 | 0.0 - 7.1 | 2.2 | 0.2 - 8.6 | 92 | 7.6 | 3.1 - 15.1 |
| 2 | 115 | 4.3 | 1.4 - 9.9 | 3.1 | 0.7 - 8.1 | 96 | 13.5 | 7.4 - 22.0 |
| 2.5 | 121 | 1.7 | 0.2 - 5.8 | 4.4 | 1.4 - 9.8 | 123 | 11.4 | 6.4 - 18.4 |
| 3 | 118 | 5.9 | 2.4 - 11.8 | 6.1 | 2.5 - 12.2 | 99 | 14.1 | 8.0 - 22.6 |
| 3.5 | 116 | 12.1 | 6.8 - 19.4 | 8.6 | 4.1 - 15.4 | 82 | 14.6 | 7.8 - 24.2 |
| 4 | 90 | 14.4 | 7.9 - 23.4 | 11.9 | 6.0 - 20.5 | 80 | 22.5 | 13.9 - 33.2 |
| 4.5 | 88 | 18.2 | 10.8 - 27.8 | 16.3 | 9.2 - 25.7 | 76 | 14.5 | 7.5 - 24.4 |
| 5 | 46 | 23.9 | 12.6 -38.8 | 21.8 | 11.0 - 36.5 | 75 | 30.7 | 20.5 - 42.4 |
| 5.5 | 39 | 23.1 | 11.1 - 39.3 | 28.6 | 15.3 - 45.4 | 82 | 30.5 | 20.8 - 41.6 |
| 6 | 29 | 34.5 | 17.9 - 54.3 | 36.6 | 19.5 - 56.6 | 74 | 36.5 | 25.6 - 48.5 |
| 6.5 | 6 | 33.3 | 4.3 - 77.7 | 43.1 | 8.0 – 85.7 | 55 | 43.6 | 30.3 - 57.7 |
| 7 | 4 | 50 | 6.8 - 93.2 | 54.4 | 7.6 - 96.2 | 24 | 58.3 | 36.6 - 77.9 |
| 7.5 | 0 |  |  |  |  | 22 | 63.6 | 40.7 - 82.8 |
| 8 | 0 |  |  |  |  | 6 | 66.7 | 22.3 - 95.7 |
