# Supplementary material for: Multidimensional Geriatric Prognostic Index, Based on a Geriatric Assessment, for Long-Term Survival in Older Adults in Korea
Source: PLoS One. 2016 Jan 15;11(1):e0147032. doi: 10.1371/journal.pone.0147032 (PMC4714804; doi:10.1371/journal.pone.0147032)
Supplement: S2 Table — (DOCX) [file pone.0147032.s002.docx]

**S2 Table**

|  | KLoSHA | | | | | SNUBH | | |
| --- | --- | --- | --- | --- | --- | --- | --- | --- |
| GPI score | Number | Observed mortality rate | 95% CI (%) | Predicted mortality rate (%) | 95% CI (%) | Number | Observed mortality rate | 95% CI (%) |
| 0 | 34 | 0 | 0.0 - 10.3 | 14.7 | 0.0 - 14.7 | 17 | 11.8 | 1.5 - 36.4 |
| 0.5 | 29 | 3.4 | 0.1 - 17.8 | 15.9 | 0.0 - 15.9 | 26 | 0 | 0.0 - 13.2 |
| 1 | 75 | 2.7 | 0.3 - 9.3 | 10.1 | 0.4 - 10.1 | 79 | 3.8 | 0.8 - 10.7 |
| 1.5 | 76 | 3.9 | 0.8 - 11.1 | 12.1 | 0.9 - 12.1 | 92 | 7.6 | 3.1 - 15.1 |
| 2 | 115 | 8.7 | 4.2 - 15.4 | 12.7 | 2.6 - 12.7 | 96 | 18.8 | 11.5 - 28.0 |
| 2.5 | 121 | 3.3 | 0.9 - 8.2 | 15.9 | 4.7 - 15.9 | 123 | 22 | 15.0 - 30.3 |
| 3 | 118 | 12.7 | 7.3 - 20.1 | 20.6 | 7.5 - 20.6 | 99 | 23.2 | 15.3 - 32.8 |
| 3.5 | 116 | 19 | 12.3 - 27.3 | 26.5 | 11.7 - 26.5 | 82 | 22 | 13.6 - 32.5 |
| 4 | 90 | 31.1 | 21.8 - 41.7 | 35.1 | 16.3 - 35.1 | 80 | 31.3 | 21.3 - 42.6 |
| 4.5 | 88 | 38.6 | 28.4 - 49.6 | 43.7 | 23.1 - 43.7 | 76 | 39.5 | 28.4 - 51.4 |
| 5 | 46 | 39.1 | 25.1 - 54.6 | 57.5 | 27.5 - 57.5 | 75 | 48 | 36.3 - 59.8 |
| 5.5 | 39 | 46.2 | 30.1 - 62.8 | 68.1 | 35.1 - 68.1 | 82 | 48.8 | 37.6 - 60.1 |
| 6 | 29 | 58.6 | 38.9 - 76.5 | 78.8 | 41.4 - 78.8 | 74 | 60.8 | 48.8 - 72.0 |
| 6.5 | 6 | 66.7 | 22.3 - 95.7 | 97.7 | 23.8 - 97.7 | 55 | 56.4 | 42.3 - 69.7 |
| 7 | 4 | 75 | 19.4 - 99.4 | 100 | 19.9 - 100.0 | 24 | 66.7 | 44.7 - 84.4 |
| 7.5 | 0 |  |  |  |  | 22 | 63.6 | 40.7 - 82.8 |
| 8 | 0 |  |  |  |  | 6 | 83.3 | 35.9 - 99.6 |
